# A microarray platform and novel SNP calling algorithm to evaluate *Plasmodium falciparum* field samples of low DNA quantity

**DOI:** 10.1186/1471-2164-15-719

**Published:** 2014-08-26

**Authors:** Christopher G Jacob, John C Tan, Becky A Miller, Asako Tan, Shannon Takala-Harrison, Michael T Ferdig, Christopher V Plowe

**Affiliations:** Malaria Group, Howard Hughes Medical Institute / Center for Vaccine Development, University of Maryland School of Medicine, Baltimore, MD 21201 USA; Research and Development, Roche NimbleGen, Inc, Madison, WI 53719 USA; Eck Institute for Global Health, Department of Biological Sciences, University of Notre Dame, Notre Dame, IN 46556 USA; Laboratory of Malaria and Vector Research, National Institute of Allergy and Infectious Diseases, National Institutes of Health, Rockville, MD 20852 USA

**Keywords:** Plasmodium falciparum, Malaria, Microarray

## Abstract

**Background:**

Analysis of single nucleotide polymorphisms (SNPs) derived from whole-genome studies allows for rapid evaluation of genome-wide diversity, and genomic epidemiology studies of *Plasmodium falciparum* provide insights into parasite population structure, gene flow, drug resistance and vaccine development. In areas with adequate cold chain facilities, large volumes of leukocyte-depleted patient blood can be frozen for use in parasite genomic analyses. In more remote endemic areas smaller volumes of infected blood are taken by finger prick, and dried and stored on filter paper. These dried blood spots do not generally yield enough concentrated parasite DNA for whole-genome sequencing.

**Results:**

A DNA microarray was designed for use on field samples to type a genome-wide set of SNPs which prior sequencing had shown to be variable in Africa, Southeast Asia, and Papua New Guinea. An algorithm was designed to call SNPs in samples with low parasite DNA. With this new algorithm SNP-calling accuracy of 98% was measured by hybridizing purified DNA from malaria lab strains and comparing calls with SNPs called from full genome sequences. An average accuracy of >98% was likewise obtained for DNA extracted from malaria field samples collected in studies in Southeast Asia, with an average call rate of > 82%.

**Conclusion:**

This new high-density microarray provided high quality SNP calls from a wide range of parasite DNA quantities, and represents a robust tool for genome-wide analysis of malaria parasites in diverse settings.

## Background

Tools for assessing genetic diversity in malaria parasites are of potential use for the discovery of novel malaria vaccine antigens [1], and for understanding the molecular basis of drug resistance [2–6]. Recent advances in genome sequencing technology offer high-throughput methods for obtaining full genomic data [7, 8]. However, these technologies still require relatively large quantities of high quality DNA. Microarrays are able to tolerate DNA of lower quantity and quality typical of patient-derived field samples, which contain far more human than parasite DNA. Thus, field samples unsuitable for full-genome sequencing may be amenable to microarray analysis. Microarrays can also be adapted to incorporate new loci, determine copy number polymorphism or designed to answer specific research questions.

Microarrays have been developed for genotyping *Plasmodium falciparum*, using a variety of platforms that detect single nucleotide polymorphisms (SNPs) at loci that had been described at the time of platform design [9–12]. Many novel SNPs were discovered in a large scale *P. falciparum* sequencing project, resulting in a map of population genomic variation [8]. We sought to create a microarray that would be able to genotype highly informative loci within this genomic map in field samples that were not amenable to next-generation sequencing. We also wanted a high-throughput system capable of working with DNA extracted from low volume blood samples. We chose to use a custom NimbleGen 4.2 million probe design in multi-plex format. This platform is comprised of 12 identical arrays on one slide, each capable of genotyping 33,716 loci within the *P. falciparum* genome. When dual-color labeling is used, two samples can be hybridized to a single array yielding 33,716 SNPs for 24 samples in a single 2-day experiment, where several such slides can be run simultaneously, making this approach relatively high-throughput and inexpensive.

## Results

### Heuristic base calling

**Figure 1:**
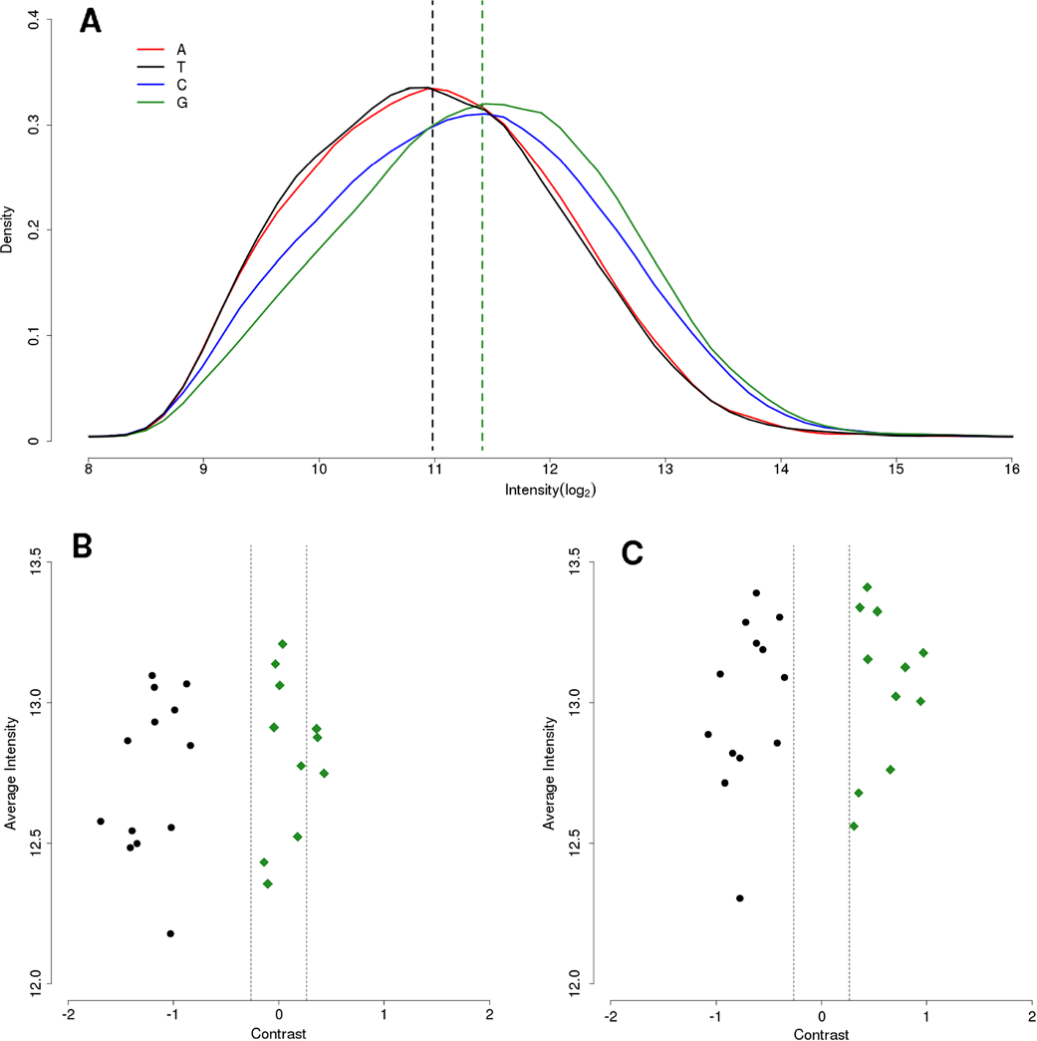
**Sample intensities and intensity distributions of varying center bases. A)** Distribution of raw probe intensities. Dotted lines indicate global mean intensity values for T (black) and G (green) center position alleles. **B)** Average raw intensities of a single SNP for 24 field samples. Dotted lines indicate area of low contrast where points between the lines would not be distinguishable. Black points were called a T allele by genome sequencing and green diamonds were called a G allele. **C)** Average intensities after global mean adjustment, all sequenced calls match microarray calls after running through algorithm.

To test the accuracy of this microarray we genotyped cultured parasites of the 3D7 reference strain, simulating varying levels of parasitemia by dilution into whole blood and using various pre- and post-extraction processing of the DNA. A previous *P. falciparum* array using the same NimbleGen technology used a SNP-calling algorithm referred to as the D-score method [9]. Applied to the new array, this approach provided an average of >92% accuracy for samples with 10,000 or more parasites/μl. However, samples with low parasitemia (<1,000 parasites/μl) yielded very poor accuracy (69% and 76%, Table 1). Investigation of this inaccuracy identified a strong bias toward G and C SNP calls in lower parasitemia samples (Figure 1A). The cause of this bias is most likely a lack of probe saturation in low DNA quantity samples and inflated intensities due to a higher binding affinity of the triple hydrogen bonding of guanine to cytosine. The D-score method relies entirely on raw intensity values to call SNPs, without regard to the base composition of the probe (Figure 1B). Many microarrays do not rely on raw intensity values to make base calls, and in some instances a complex model is fitted to the data to accurately call SNPs [13, 14]. To overcome this bias a heuristic algorithm was developed which uses the probe center base position to adjust raw intensity values (Figure 1C). When applied to the samples described above, this new algorithm gave accuracies greater than 95% in nearly all samples tested.

**Table 1:** **SNP call accuracy and SNP call rate of 3D7 cultured parasites**

	SNP call accuracy								SNP call rate							
	Heuristic algorithm				D-score method				Heuristic algorithm				D-score method			
WGA	+		-		+		-		+		-		+		-	
LD	+	-	+	-	+	-	+	-	+	-	+	-	+	-	+	-
1,000 p/μl	99.0	96.2	99.6	94.1	93.5	76.4	97.4	69.0	75.4	36.8	68.7	15.6	56.0	40.7	63.2	32.3
10,000 p/μl	99.4	99.4	99.7	99.5	94.4	95.8	98.6	94.1	76.5	87.5	87.6	62.9	51.7	59.1	70.8	50.4
100,000 p/μl	-	99.6	-	99.7	-	98.7	-	98.8	-	94.4	-	92.0	-	76.0	-	78.9
500,000 p/μl	-	99.7	-	99.8	-	98.4	-	98.9	-	94.5	-	95.3	-	64.4	-	72.8

To test the accuracy and call rate of the new heuristic algorithm cultured 3D7 strain parasites were genotyped under differing leukocyte depletion, whole-genome amplification, and parasite concentrations. We then compared the previously describe D-score method to the heuristic algorithm. (WGA = whole-genome amplification, LD = leukocyte depletion, p/μl = parasites per microliter).

This algorithm uses the global array average for probes with identical center positions to adjust individual probe intensities. A call is made only when the sense and antisense base match, a contrast between alleles of 0.98 exists, and all intensities are greater than the random binding threshold. This heuristic algorithm outperformed the D-score method in the mixing experiment of cultured 3D7 parasites raising the average accuracy of these samples to >98% and increasing the accuracy of the 1,000 parasites/μl by as much as 25%. We also observed an increase in SNP call rates when using this heuristic algorithm.

**Figure 2:**
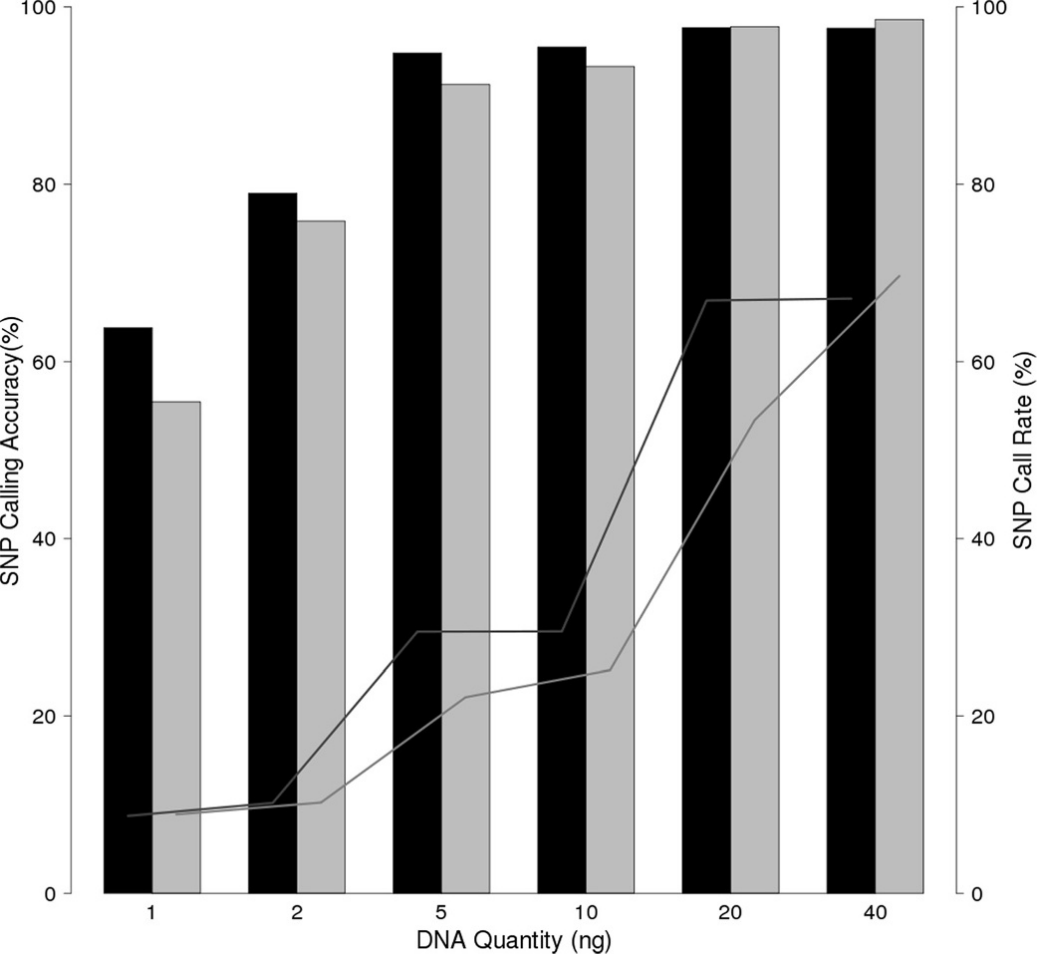
**SNP call accuracy and SNP call rate of low quantity reference DNA.** Black and grey bars show SNP accuracy of purified 3D7 and Dd2 strain parasites at low DNA quantities (1 – 40 ng). Lines show SNP call rate of each typed sample. DNA quantity was significantly associated with SNP calling accuracy (p = 0.025), and DNA quantity with SNP call rate (p = 1.57e-05).

To set more accurate sample quality control (QC) thresholds we switched from the initial approach of measuring DNA quantity in terms of parasites/μl to a quantitative PCR (qPCR) method. For the previous microarray a DNA quantify cutoff of 250 ng was used to select samples for testing, but lower input amounts were not systematically evaluated [9]. Adopting the new heuristic algorithm and using 152–219 ng DNA from the NF54 parasite lab strain quantified using 18 s qPCR, we observed an average SNP-calling accuracy of 99.4% (Table 2). To identify the lower limits of DNA quantity we tested a range of 1–40 ng purified DNA from 3D7 and Dd2 parasites, obtaining >95% accuracy down to 20 ng of parasite DNA for 3D7 and down to 10 ng for Dd2. Calculation of call rates for all samples revealed a wide range (8% - 70%) which was associated with the amount of DNA used (p = 1.57e-05) (Figure 2). For these samples there was also an association of DNA quantity with call accuracy (p = 0.025).

**Table 2:** **SNP call accuracy and SNP call rate of NF54 purified DNA**

ID	Parasite DNA (ng)	Call accuracy	Call rate
NF54-1	219	99.4%	86.6%
NF54-2	212	99.5%	81.5%
NF54-3	210	99.4%	78.6%
NF54-4	203	99.4%	73.8%
NF54-5	170	99.4%	85.7%
NF54-6	168	99.4%	77.3%
NF54-7	156	99.2%	76.9%
NF54-8	152	99.3%	69.0%
NF54 (WGA)	222	99.6%	91.6%
NF54 (WGA)	138	99.5%	85.1%

NF54 reference strain DNA was typed at varying concentrations and call accuracy and call rate was calculated. Two samples underwent whole genome amplification (WGA) prior to genotyping and the concentration reflects post-WGA quantities.

### Analysis of field isolates and leukocyte depletion

**Figure 3:**
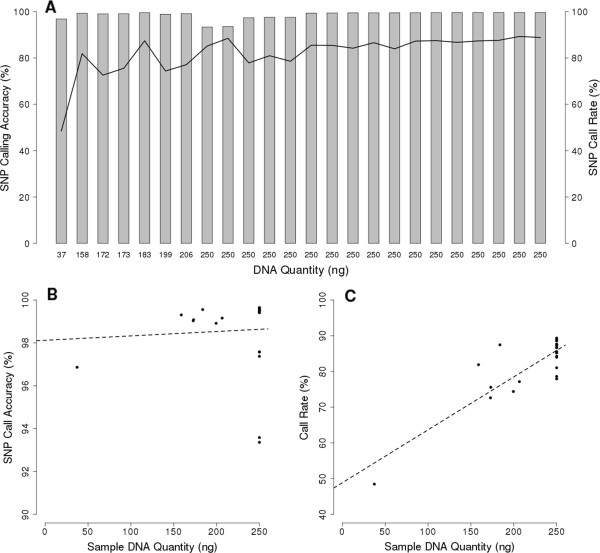
**SNP call accuracy and SNP call rate of field samples. A)** SNP calling accuracy (grey bars) and SNP call rates (black line) for 24 field isolate from Southeast Asia. **B)** Correlation of SNP call accuracy and DNA quantity (p = 0.79). **C)** Correlation of SNP call rate with DNA quantity (p = 9.8e-08).

To assess the feasibility and accuracy of using DNA from *P. falciparum* parasites extracted directly from patient blood we used both high and low parasite DNA concentration samples derived from venous blood samples that had and had not been subjected to leukocyte depletion. High DNA samples include 17 samples from Southeast Asia that had successfully undergone Illumina whole-genome sequencing and had a parasite DNA concentration of 250 ng. These samples had an average accuracy of 98.4% and an average call rate of 85.4% (Figure 3A). Low DNA volume samples were seven samples from Southeast Asia that ranged from 206 ng down to 37 ng of parasite DNA and averaged 98.8% and 73.9% accuracy and call rate respectively. There was no correlation between accuracy and DNA quantity (p = 0.79) (Figure 3B), while there was a strong correlation (p = 9.8e-08) (Figure 3C) between DNA quantity and call rate, demonstrating that DNA quantity is a good predictor of the number of SNPs called. Analyses were performed on all reference and field isolates tested to determine the per SNP accuracy rate. Among all 33,716 SNPs typed by this array ~28,000 were called correctly in 100% of samples analyzed. Only a small subset (~200) was called incorrectly in all samples and ~600 were not able to be called in any sample. Only SNPs called correctly in >95% of samples were included in final versions of the heuristic base calling algorithm. Distribution of inaccurate SNPs appears random through visual inspection of both their place within the genome and their layout on the printed array.

We noted a difference for low volume samples that had undergone leukocyte depletion to remove human DNA prior to DNA extraction. Leukocyte depletion removes human white blood cells from a whole blood sample, enriching the final sample for red blood cells which harbor the parasite and consequently their DNA. In the analysis of cultured 3D7 parasites diluted in whole blood a subset of samples were leukocyte depleted using CF11 cellulose columns. For the 1,000 parasites/μl samples we saw an average increase of accuracy of 4.2% and an average increase in SNP call rate of 45.9% (Table 1). This increase was more pronounced in non-whole genome amplified (WGA) samples.

### Effects of whole-genome amplification

Samples with low DNA quantity can be subjected to WGA to increase the amount of DNA, allowing more samples to pass preliminary QC metrics. Several cultured and purified DNA samples underwent WGA prior to genotyping and were used to evaluate possible bias when undergoing amplification. Accuracies in cultured parasites showed no marked difference (≤0.2%) while WGA slightly increased the call rate overall (Table 1). Two NF54 DNA samples were subjected to WGA prior to microarray analysis, yielding an average accuracy of 99.54% and an average call rate of 88.34%, about 10% higher than the non-WGA samples (Table 2).

## Discussion

We set out to develop a SNP genotyping platform for use on *P. falciparum* field samples of low DNA quantity and quality, and evaluated this tool using parasite DNA from cultured parasites and preserved venous blood collected from malaria-infected individuals in field studies. An earlier microarray used 250 ng of DNA as the minimum level tested, although lower DNA quantities were not systematically evaluated [9]. Our heuristic SNP-calling algorithm was capable of accounting for a bias discovered when using lower quantity samples and yields highly accurate results for samples with as little as 37 ng of parasite DNA in field samples and 10 ng in purified DNA samples. We also obtained high accuracy with filter paper samples, albeit with lower reproducibility (not shown). Addition of a WGA step and a strict DNA quantity threshold may improve reproducibility for filter paper blood spots.

DNA microarrays have previously been used for SNP detection in *P. falciparum* [9–11, 15] *.* With the increased availability of whole genome sequence data thanks to lower costs and improved methodology, the value of DNA microarrays can be questioned. However, for valuable field samples that are associated with important demographic, clinical and other phenotypic data such as drug resistance or vaccine resistance [16], microarrays can rescue the high proportion of samples that fail to meet criteria for sequencing, justifying the use of microarrays. Samples that often do not meet sequencing standards include low volume blood samples, filter paper blood spots, samples with high levels of human DNA contamination, and archived or degraded samples. It was our goal to create a SNP microarray that can complement genome sequence data in association and population genetics studies, providing genome-wide SNP data for samples not fit for sequencing. The SNPs chosen were based on a set of highly validated loci identified by sequencing hundreds of global isolates of *P. falciparum* [8] *.* This large sequencing project shows how microarrays still have a place in genomic research. The first iteration of samples prepared for sequencing had a QC pass rate of ~30%; more recent samples had a pass rate of >50%. This leaves a significant number of samples with no data. By selecting SNPs from within this highly validated list of loci we can fill in the gaps from sequencing while continuing to use samples for which sequencing was successful. The cost of genome-wide microarray analysis (currently less than $90/sample excluding personnel costs) compares favorably with that of genome sequencing (at least 3-fold higher for short-read Illumina sequencing and approximately 25-fold higher for third generation sequencing and assembly). Imputation strategies are being developed that may further increase the value of microarray-generated SNP information.

## Conclusions

This microarray genotypes over 33,000 variable positions in the *P. falciparum* genome with high accuracy and high throughput. The ability to run samples with as little as 10 ng of parasite DNA increases the number of field samples for which whole genome analysis is possible. The selection of loci also allows samples genotyped on this microarray to be analyzed in conjunction with higher quality samples sequenced using next-generation sequencing platforms.

## Methods

### SNP selection and chip design

A pooled set of all possible SNPs was gathered from Version 1.0 of the MalariaGEN Community Project [8] and a SNP set from an Affymetrix array used in a previous study [3]. This pooled set was filtered based on the proportion of missing data in previous data sets, hyper-heterozygosity, and minor allele-frequency (MAF) in Southeast Asia and Africa ≥1%. SNPs were prioritized by their MAF with precedence given to those with higher MAF values. SNPs given the highest priority were those that were variable in multiple populations. SNPs were ranked in order of priority and then a final filter of minimum inter-SNP distance was applied. To decrease the number of large genomic gaps lower priority SNPs were given preference to high priority SNPs that were within 22 bp of another high priority SNP. The 22 bp value was determined through systematically increasing the inter-SNP distance threshold and measuring the number of large genomic gaps and total number of loci.

**Figure 4:**
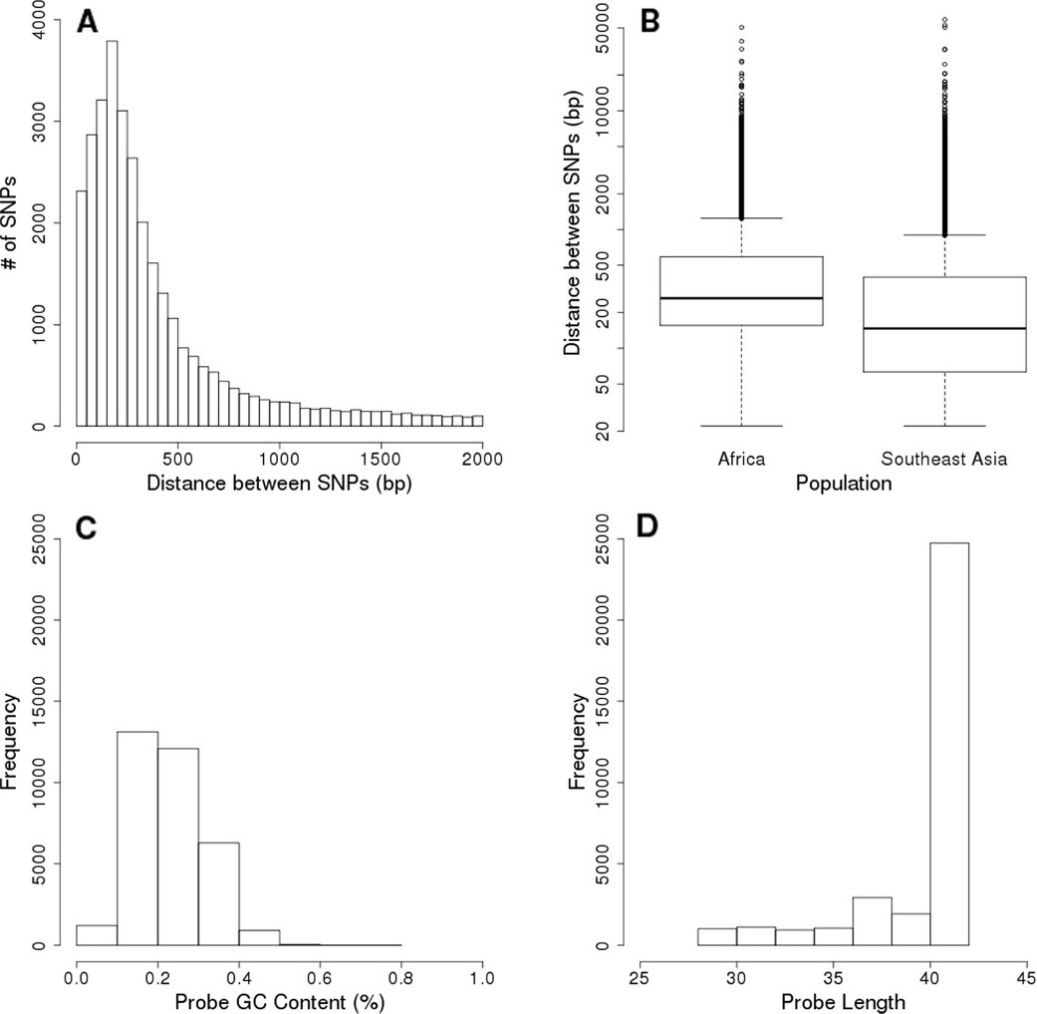
**Microarray probe statistics. A)** Histogram of Inter-SNP distances of all SNPs. **B)** Boxplot showing distribution of inter-SNP distances where allele variability has been shown. **C)** Histogram of G + C content of probes. **D)** Histogram showing distribution of probe lengths.

SNPs from African and Southeast Asian parasites were chosen to provide full coverage of the *P. falciparum* genome (minus telemetric regions and hyper-variable *var* gene clusters). Among the SNPs chosen for the array, 22,189 were variable only in Africa, 12,723 in Southeast Asia, and 3,811 in Papua New Guinea. Among the SNPs variable in Africa and Southeast Asia 6,007 were variable on both continents. Also included on the array were 23 loci associated with drug resistance in the genes *pfcrt, pfmdr-1, pfdhps,* and *pfdhfr*. The entire SNP set averaged a median per chromosome inter-SNP distance of 324 bp (Figure 4A).

The probes designed for the array were mostly low GC content (Figure 4C) and of variable lengths (29–41 bp) (Figure 4D). Variable length probes provide higher average intensities when compared to static length probes and low GC was due to the genomic AT richness of the *P. falciparum* genome [9]. Standard NimbleGen probes were also placed on the array, including alignment probes, chip identification probes, and random cDNA probes designed to match the content of our user designed probes. The average intensity of 8,421 random probes was used to define the global array background.

The NimbleGen 4.2 M Probe Custom DNA Microarray comes in varying formats from which we selected the highest number of plexes (12) to increase the number of samples for use in high-throughput fields studies. Probe length was determined by cDNA melting temperature of the sequence surrounding each SNP. For every SNP there were eight probes, four in the sense direction with each base at the center position and four in the anti-sense direction. Probe quartets were arranged sequential on the array to avoid variation due to chip defects and spatial bias. This array is no longer available as NimbleGen has discontinued custom microarray production. Steps are being taken to transfer this probe set and analysis pipeline to a new commercial platform.

### DNA labeling and hybridization

Sample DNA was concentrated using vacuum centrifugation to a volume of 30–50 μl and heat denatured with 1 OD of 65% random nonamers labeled with cy3 or cy5 for 10 minutes at 98°C. Denatured DNA was chilled on ice for 2 minutes and then incubated for 2 hours at 37°C in the presence of 50 units of Klenow fragment and a 50X dNTP mixture. The reaction was terminated with 0.5 M EDTA and DNA were precipitated with 5 M NaCl and iso-propanol. Labeled DNA was washed 2–3 times with 80% ice cold ethanol to remove unincorporated dye. After removal of ethanol, samples were rehydrated in water and cy3 and cy5 labeled samples were combined for multiplexing. Samples were vacuum-concentrated and resuspended in a buffer containing NimbleGen alignment oligo and 1X Denhardt’s solution. The samples were heat denatured at 95°C for 5 minutes and stabilized at 42°C prior to loading onto the array. Samples were hybridized on a NimbleGen hybridization station for 16–24 hours at 42°C. Slides were disassembled in a dish containing Wash Buffer 1 at 42°C and washed in Wash Buffer 1, Wash Buffer 2, and Wash Buffer 3 for 2 minutes, 1 minute, and 15 seconds respectively. Slides were washed and subsequently dried in the SlideWasher 12 Array Processing System. Microarrays were scanned with a NimbleGen MS 200 Microarray Scanner at 2 μm using “autogain” to automatically adjust scanning parameters on an individual array basis.

### Heuristic base calling algorithm

Each SNP typed by this array is at a bi-allelic locus, as determined from extensive sequencing of global *P. falciparum* isolates [8]. The heuristic algorithm therefore focuses on the two intensities of each possible allele. This algorithm then identifies the global mean intensity for every probe with the identical center base and adjusts each individual intensity by the difference in intensity between the global means of the two bases being interrogated. Intensities are evaluated and adjusted independently for the sense and anti-sense direction. After adjusting the intensity, a SNP is called after fulfilling the following criteria: 1) the contrast of intensities is greater than or equal to 0.98, 2) the forward and reverse SNP calls are concordant, and 3) all intensities are above global background levels determined by the average value of the random probes. Multiple thresholds were tested for background levels up to average random plus two standard deviations (data not shown). SNP calling accuracy changed minimally when thresholds were raised, however SNP call rate dropped more significantly. This algorithm was written in the PERL programming language and uses standard outputs from the Roche NimbleScan (v2.6) software. Given the discontinuation of this microarray the algorithm will be made publically available when the probe set is validated on a new platform.

### Experimental samples

*P. falciparum* (3D7 strain) was grown in culture under standard conditions from stocks procured from the MR4 repository. Base parasitemia was determined by light microscopy and dilutions were made using human whole blood. Dilutions were made to simulate 1,000, 10,000, 100,000, and 500,000 parasites/μl. Leukocyte depletion was performed on a subset of samples using 2.5 mL of blood in a CF11 column. Insufficient parasites were acquired to fulfill the necessary 2.5 mL of blood for leukocyte depletion of the 100,000 and 500,000 parasites/μl mixture. DNA extraction of these samples was performed with the Qiagen mini-kit. Dd2 and 3D7 purified DNA was also received from the MR4 repository. Purified NF54 parasite DNA was generously donated by Sanaria Inc. in Rockville MD. Whole blood samples from field isolates came from studies conducted in Southeast Asia [Takala-Harrison *et al.* under review]. A subset of samples was whole-genome amplified prior to experimentation, for these samples we used a Qiagen REPLI-g mini kit. Simple linear regression was used to test the relationship of DNA quantity with SNP call rate and SNP call accuracy. Informed consent was obtained from participants or their parents or guardians for samples collected as part of clinical trials following protocols approved by the Research Ethics Review Committee of the World Health Organization, local Institutional Review Boards (IRBs), and samples were analyzed following a protocol approved by the University of Maryland, Baltimore IRB.

### Quantitative PCR

The *P. falciparum* gene encoding the 18 s ribosomal subunit was amplified using qPCR for each sample. In a total reaction volume of 25 μl, 2 μl of sample DNA was used along with 10 μM probe, 10 μM of each primer, water, and TaqMan Universal PCR Master Mix (containing AmpliTaq Gold DNA Polymerase, dNTPs, and dUTP). The sequences for primers and probe follows: Forward- 5′-GTA ATT GGA ATG ATA GGA ATT TAC AAG GT-3′, Reverse- 5′-TCA ACT ACG AAC GTT TTA ACT GCA AC-3′, Probe- 5′-FAM GAA CGG GAG GTT AAC AA MGB-3′. PCR conditions were 15 minutes at 95°C, 15 seconds at 95°C, and 45 cycles for 1 minute at 60°C. For quantification a standard curve was generated and run on each plate as well as a no DNA control. Standard curve DNA was derived from purified NF54 parasite DNA and quantified using CYBR Green. This DNA was diluted to 3, 1.5, 0.75, 0.375, 0.188, 0.094, and 0.047 ng per μl and each standard and sample was run in duplicate with the final quantity expressed as the mean of both values.

### Availability of supporting data

The data set supporting the results of this article is available in the NCBI Gene Expression Omnibus data repository, Accession: GSE56305, http://www.ncbi.nlm.nih.gov/geo/.

## Abbreviations

SNP: Single nucleotide polymorphism
qPCR: Quantitative polymerase chain reaction
WGA: Whole-genome amplification
QC: Quality control.
